# T cell dysfunction and metabolic disruption in chronic hepatitis C virus infection

**DOI:** 10.3389/fimmu.2026.1844075

**Published:** 2026-06-01

**Authors:** Bhavya Sajeet, Uma Ganapathi, Kiruthika Naganathan, Aarthi Parthasarathy, Pramod Darvin, Varun Sasidharan Nair

**Affiliations:** 1 Department of Biochemistry, Mahalashmi Women’s College of Arts and Science, Chennai, India; 2 Centre for Marine Science and Technology, Manonmaniam Sundaranar University, Rajakkamangalam, India; 3 Department of Microbiology, Saveetha Institute of Basic Medical Sciences (SIBMS) Saveetha University, Chennai, Tamil Nadu, India; 4 Department of Tumour Biology, Barts Cancer Institute, Queen Mary University of London, London, United Kingdom; 5 Genetics and Epigenetics of Behavior Laboratory, Fondazione Istituto Italiano di Tecnologia (IIT), Genoa, Italy

**Keywords:** epigenetic scarring, hepatitis C virus, mitochondrial dysfunction, T cell exhaustion, regulatory T cells

## Abstract

Hepatitis C virus (HCV) infection remains a major global health burden and a leading cause of chronic liver disease, cirrhosis, and hepatocellular carcinoma. Despite the availability of highly effective direct-acting antivirals, sustained immune dysfunction and long-term complications continue to challenge disease management. Chronic HCV infection is facilitated by multiple viral evasion mechanisms, including rapid sequence variation, disruption of innate antiviral signaling, and altered natural killer cell function. A key feature of disease progression is the dysfunction of virus-specific CD4^+^ and CD8^+^ T cells caused by prolonged antigen exposure. These cells gradually develop an exhausted phenotype marked by reduced proliferation, impaired cytokine production, and increased expression of inhibitory receptors such as PD-1, CTLA-4, TIM-3, and TIGIT. At the same time, intrahepatic accumulation of regulatory T cells further suppresses antiviral immune responses and promotes viral persistence. Recent studies also show that chronic HCV infection induces significant metabolic and mitochondrial dysfunction including oxidative stress, impaired bioenergetics, and altered glycolytic adaptation, all of which contribute to defective T cell responses and disease progression. Notably, some of these immune defects persist even after viral eradication because of stable transcriptional and epigenetic changes in exhausted T cells. This review summarizes current understanding of how T cell dysfunction, epigenetic programming, and metabolic disruption interact in chronic HCV infection. Understanding these interconnected mechanisms may guide the development of novel therapeutic strategies that combine antiviral, immunomodulatory, and metabolic interventions to achieve durable immune restoration and improved clinical outcomes.

## Introduction

Hepatitis C virus (HCV) infection remains a major global public health challenge and a leading cause of chronic liver disease worldwide. HCV is an enveloped, positive-sense single-stranded RNA virus of the *Flaviviridae* family and has been recognized for decades as a leading cause of progressive liver injury (1, 2). Although the introduction of direct-acting antivirals (DAA) has greatly improved treatment outcomes and made viral eradication possible in most patients, HCV continues to impose a substantial clinical and public health burden due to delayed diagnosis, silent early infection, and long-term complications associated with chronic liver disease (3, 4) including immunological abnormalities. As a result, HCV remains a relevant challenge in both high-resource and low-resource settings, requiring continued attention to its virology, immunopathogenesis, and long-term clinical consequences.

Recent epidemiological analyses from the Global Burden of Disease 2021 study highlights this persistent global burden, reporting 11.64 million new incidence alongside considerable mortality, prevalence, and disability-adjusted life years (5). A major obstacle to HCV control is that acute infection is often asymptomatic or clinically mild, diagnosis is frequently delayed until advanced liver injury has developed, which obscures the full extent of the viral reservoir and complicates efforts to achieve the World Health Organization’s 2030 elimination targets (6). This hidden disease burden is further sustained by ongoing transmission through exposure to infected blood, including unsafe medical procedures, transfusion of unscreened blood products, and injection drug use, while vertical and sexual transmission occur less commonly (5, 7). The relative importance of these routes varies across regions and reflects differences in healthcare infrastructure, blood safety, harm-reduction access, and public health capacity (8). Together these factors continue to hinder case finding, early treatment, and progress toward global HCV elimination.

The clinical course of HCV infection is strongly influenced by an interaction between viral persistence and host immune responses. A relative minority (20-30%) of infected individuals spontaneously clear the virus, usually through coordinated and effective innate and adaptive immune responses, whereas the majority (50-85%) develop chronic infection with ongoing viral replication and progressive liver damage (9, 10). This persistence is facilitated by multiple immune evasion mechanisms, rapid genetic variation, interference with innate immune sensing and interferon signaling, and functional impairment of antiviral lymphocyte responses (11, 12). Continuous antigenic exposure further drives immune dysfunction, particularly T cell exhaustion, which weakens viral control and promotes chronic inflammation (13). These processes place immune dysregulation at the core of HCV pathogenesis and help explain differences in viral clearance, disease progression, and long-term outcomes among infected individuals.

Innate immunity provides the first line of defense against HCV infection and plays a critical role in the early control of viral replication. Following HCV infection, viral RNA is recognized by pattern-recognition receptors in hepatocytes and innate immune cells, including RIG-I-like receptors and TLR3, which initiate antiviral signaling and promote downstream activation of effector populations such as natural killer cells (11). Activation of these receptors triggers signaling pathways that induce type I and type III interferons and other pro-inflammatory mediators (11). These early antiviral responses help restrict viral replication and helps in the later adaptive immune response. Natural killer (NK) cells are major effector cells in this phase and contribute to antiviral defense through both cytotoxic and non-cytolytic mechanisms, including perforin- and granzyme-mediated killing of infected cells and production of interferon (IFN)-γ (9, 11). However, in chronic HCV infection, innate immune responses are frequently delayed, incomplete, or functionally altered. NK cells may show abnormal receptor expression, reduced cytokine production, and impaired coordination with other immune populations, while viral proteins can interfere directly with intracellular antiviral signaling pathways. Together, these defects weaken early viral control and promote an intrahepatic environment that supports viral persistence and ongoing liver inflammation (9, 11, 14).

Adaptive immunity plays a decisive role in determining the outcome of HCV infection and effective cellular immune responses are central to viral clearance (9, 15). Although humoral immunity contributes through the production of neutralizing antibodies that can limit viral entry, T cell responses are particularly important in controlling infection. CD8^+^ cytotoxic T lymphocytes recognize viral peptides presented by major histocompatibility complex (MHC) class I molecules and eliminate infected hepatocytes through perforin- and granzyme-mediated cytotoxicity, while also producing antiviral cytokines such as IFN-γ and tumor necrosis factor (TNF)-α (15, 16). CD4^+^ T helper cells support these responses by promoting CD8^+^ T cell expansion, sustaining effector function, and facilitating B-cell maturation and antibody production. These cells differentiate into functionally distinct subsets, including Th1, Th2, Th17, follicular helper T cells (Tfh), and regulatory T cells (Tregs), each of which contributes differently to antiviral immunity and immune regulation (13). In particular, Th1 responses promote antiviral cellular immunity whereas regulatory T cells help maintain immune homeostasis but may also suppress protective responses during chronic infection. During persistent antigen exposure, both CD4^+^ and CD8^+^ T cells gradually lose their antiviral function and acquire an exhausted phenotype. This state is marked by reduced proliferation, lower cytokine production, and increased expression of inhibitory receptors such as PD-1, CTLA-4, and TIM-3. Together, these changes weaken antiviral immunity and promote viral persistence (12, 13).

Overall, the pathogenesis of HCV infection reflects a complex interaction between viral replication mechanisms and host immune responses. Although both innate and adaptive immunity contribute to antiviral defense, multiple immune evasion strategies enable HCV to establish chronic infection. Dysfunction of CD4^+^ and CD8^+^ T cell responses is a central feature of this process and plays a major role in viral persistence, chronic inflammation, and progressive liver injury. Understanding the mechanisms that drive T cell impairment including exhaustion, epigenetic reprogramming, and metabolic disruption, is therefore important for improving current therapeutic approaches (17, 18). In this review, we summarize current knowledge on T cell dysfunction in chronic HCV infection, with particular emphasis on the links between immune exhaustion and metabolic dysregulation and discuss how these insights may inform future antiviral and immunomodulatory strategies.

## HCV replication dynamics and immune modulation

HCV establishes persistent infection through a tightly coordinated replication cycle that not only enables efficient viral propagation but also shapes host immune responses, particularly T cell function. Viral entry into hepatocytes is mediated by envelope glycoproteins E1 and E2 through sequential interactions with host receptors including scavenger receptor class B type I (SR-BI), CD81, claudin-1, and occludin, leading to clathrin-mediated endocytosis and release of the viral RNA genome into the cytoplasm (19, 20). Following entry, the positive-sense RNA genome is directly translated at the endoplasmic reticulum via an internal ribosome entry site, producing a polyprotein that is cleaved into structural and non-structural proteins essential for replication (21).

HCV replication occurs within specialized endoplasmic reticulum-derived membranous webs composed of double-membrane vesicles, which concentrate viral proteins and replication intermediates while limiting exposure of viral RNA to cytosolic pattern recognition receptors such as RIG-I and MDA5 (22, 23). This spatial sequestration represents a key immune evasion mechanism, enabling sustained antigen production and chronic immune stimulation. Sustained antigen availability drives continuous T cell receptor engagement, which promotes progressive exhaustion of virus-specific CD4^+^ and CD8^+^ T cells (24). In addition to this compartmentalization of viral replication, HCV further modulates host antiviral responses through the activity of viral proteins. The NS3/4A protease disrupts innate immune signaling by cleaving adaptor molecules involved in interferon induction, thereby weakening downstream antiviral responses and promoting viral persistence (25). In parallel, NS5A acts as a key immunomodulator that disrupts both innate and adaptive immune pathways, including PKR- and PI3K-associated signaling, thereby suppressing antiviral responses and limiting immune-mediated clearance of infected hepatocytes (26). Moreover, NS5A constrains CD8^+^ T cell functionality by reducing polyfunctionality and cytotoxic capacity under immune-tolerant conditions. Together with NS3/4A-mediated inhibition of innate signaling, these mechanisms impair T cell priming and effector function, promoting viral persistence (26).

Importantly, viral replication actively shapes the magnitude and quality of T cell responses. Experimental models demonstrate that IFN-γ secretion mediates potent inhibition of viral RNA replication independently of target cell lysis, as >95% suppression can occur at low effector-to-target ratios without detectable cytotoxicity (27). This indicates that antiviral control is primarily driven by cytokine-mediated mechanisms rather than cell killing. Consequently, the effectiveness of viral suppression is determined by CD8^+^ T cell functional capacity, particularly cytokine production, rather than cell number, indicating that functional impairment of these cells contributes directly to viral persistence. In addition to functional capacity, the effectiveness of T cell-mediated control is further determined by antigen specificity and T cell receptor (TCR) avidity. High-avidity T cells targeting conserved viral epitopes, particularly within NS3, exhibit enhanced recognition of infected hepatocytes, including cells with low antigen presentation, and are consistently associated with viral clearance (28). Such high-avidity TCR interactions enable efficient detection of viral variants and sustained immune constraint on replication. In contrast, failure to maintain such high-avidity responses reduces the efficiency of target recognition and antiviral activity, thereby allowing continued viral replication and progression to chronic infection (28). At a broader level, viral replication and T cell function are mechanistically coupled through antigen-dependent signaling. Persistent high-level viremia maintains continuous antigen exposure, which drives loss of functional HCV-specific CD4^+^ T cell responses during chronic infection. Conversely, reductions in viral replication decrease antigenic stimulation, enabling partial restoration of CD4^+^ T cell immunity (29). Clinical evidence shows that declines in viremia are associated with increased frequencies of HCV-specific CD4^+^ T cells with effector-memory phenotype and restored Th1 functionality, including IL-2 and IFN-γ production (29). These findings demonstrate that antigen burden directly regulates CD4^+^ T cell functional capacity and that T cell dysfunction is at least partially reversible and dependent on ongoing viral replication.

HCV replication also alters host metabolic pathways in a manner that indirectly constrains T cell function. Viral dependence on host lipid metabolism and bioenergetic pathways promotes metabolic reprogramming within the liver microenvironment (30). In this context, persistent antigen exposure and co-inhibitory receptor signaling further impose T cell-intrinsic metabolic defects. During chronic infection, sustained TCR stimulation and upregulation of co-inhibitory receptors such as PD-1 and CTLA-4 suppress PI3K/AKT/mTOR signaling, reduce glucose uptake and glycolysis, and impair mitochondrial function. These metabolic constraints limit T cell activation, cytokine production, and differentiation into effector and memory populations (31). Thus, metabolic dysfunction acts as an upstream driver that reinforces and stabilizes T cell exhaustion rather than merely resulting from it.

Although DAAs effectively suppress viral replication by targeting NS3/4A, NS5A, and NS5B, elimination of viremia does not uniformly restore immune competence (32). While viral suppression reduces antigenic stimulation, persistent defects in T cell function often remain, reflecting durable transcriptional, epigenetic, and metabolic alterations established during chronic infection. These observations highlight that effective management of HCV requires not only antiviral suppression but also strategies aimed at restoring immune function, particularly T cell competence.

## Innate and interferon-mediated immune responses in hepatitis C virus infection

The innate immune response plays a central role during early HCV infection, particularly within hepatocytes. Following viral entry and replication, host cells detect viral RNA through pattern recognition receptors (PRRs), initiating antiviral signaling that restricts replication and shapes downstream immune responses (33). The strength and timing of these responses are critical in determining whether infection is cleared or progresses to chronicity. PRR activation triggers signaling cascades that culminate in type I interferon (IFN) production and the induction of interferon-stimulated genes (ISGs), which collectively inhibit viral replication and enhance antigen presentation, thereby linking innate and adaptive immunity (9, 33, 34). Despite this coordinated response, HCV frequently induces delayed or incomplete IFN production, facilitating viral persistence (35).

**Figure 1:**
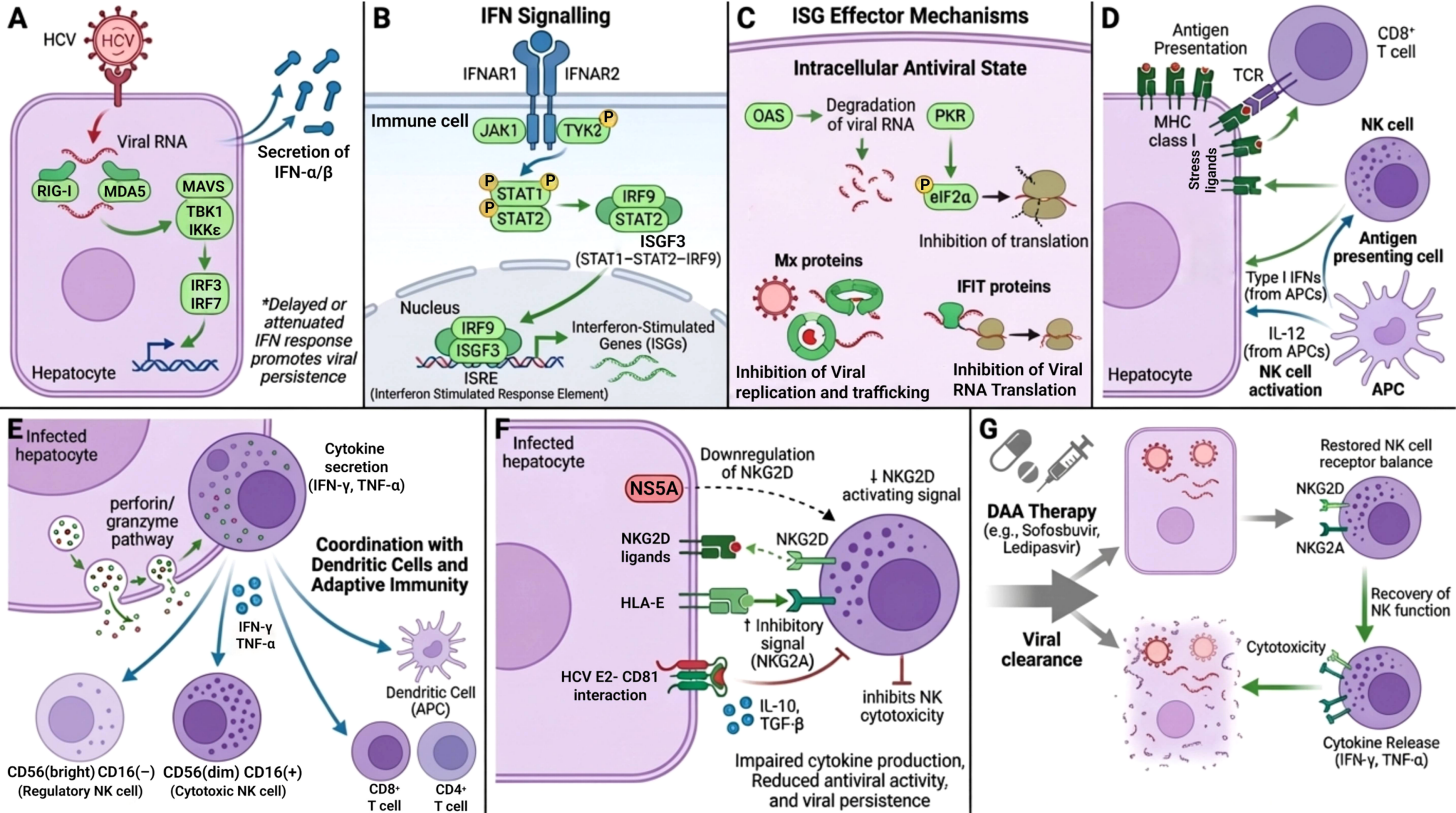
Interferon signaling and NK-cell-mediated antiviral responses in HCV infection. **(A)** HCV infection of hepatocytes is sensed by the cytosolic RNA sensors RIG-I and MDA5, which signal through MAVS, TBK1, and IKKϵ to activate IRF3/IRF7 and induce secretion of type I interferons (IFN-α/β); delayed or attenuated IFN responses favor viral persistence. **(B)** Type I IFNs engage IFNAR1/IFNAR2 on immune cells, activating JAK1 and TYK2, which phosphorylate STAT1 and STAT2 to form the ISGF3 complex with IRF9; ISGF3 translocates to the nucleus, binds interferon-stimulated response elements (ISREs), and induces interferon-stimulated genes (ISGs). **(C)** ISG effector pathways establish an intracellular antiviral state through OAS-mediated viral RNA degradation, PKR-dependent eIF2α phosphorylation and translational arrest, and antiviral actions of Mx and IFIT proteins that inhibit viral replication, trafficking, and RNA translation. **(D)** Antigen-presenting cells (APCs) promote antiviral immunity by producing type I IFNs and IL-12, enhancing NK-cell activation and facilitating crosstalk between stressed hepatocytes, NK cells, and CD8^+^ T cells through antigen presentation and stress-ligand recognition. **(E)** Activated NK cells contribute to antiviral defense by killing infected hepatocytes through perforin/granzyme pathways, secreting IFN-γ and TNF-α, and coordinating with dendritic cells and adaptive immune cells, including CD4^+^ and CD8^+^ T cells. **(F)** During chronic infection, HCV impairs NK-cell function through NS5A-mediated downregulation of NKG2D signaling, HLA-E/NKG2A inhibitory signaling, HCV E2-CD81 interactions, and IL-10/TGF-beta-mediated suppression, resulting in reduced cytokine production, impaired cytotoxicity, and viral persistence. **(G)** Direct-acting antiviral (DAA) therapy promotes viral clearance and partially restores NK-cell receptor balance and effector function, including cytotoxicity and cytokine release.

Interferon signaling represents a central antiviral defense pathway during HCV infection (**Figure 1**). Viral RNA recognition leads to the production of type I interferons, which bind to the interferon-α/β receptor (IFNAR1/IFNAR2) on target cells. This interaction activates the JAK-STAT signaling pathway, involving Janus kinase 1 (JAK1) and tyrosine kinase 2 (TYK2), which phosphorylate STAT1 and STAT2 (36). Phosphorylated STAT1 and STAT2 form a complex with interferon regulatory factor 9 (IRF9), known as ISGF3. This complex translocates to the nucleus, where it binds interferon-stimulated response elements (ISREs) and induces transcription of ISGs (**Figure 1**) (36). ISGs encode a diverse range of antiviral proteins that target multiple stages of the viral life cycle. Among these, several well-characterized ISGs mediate direct antiviral effects through distinct molecular mechanisms (34). The 2′,5′-oligoadenylate synthetase (OAS)-RNase L pathway degrades viral RNA, protein kinase R (PKR) inhibits translation through phosphorylation of eIF2α, Mx GTPases disrupt viral replication and transport, and IFIT proteins block translation of viral RNA **Figure 1**) (34). Through these coordinated actions, interferon signaling establishes a strong intracellular antiviral state and contributes to the early containment of HCV infection.

In addition to direct antiviral effects, interferon signaling enhances immune responses by upregulating MHC class I expression, thereby improving antigen presentation to cytotoxic T lymphocytes (37). Furthermore, interferons play a key role in linking innate immune signaling to cellular effector responses by promoting activation of natural killer (NK) cells. These liver-enriched cytotoxic lymphocytes are activated by infected hepatocytes, antigen-presenting cells, and cytokines such as type I IFNs and IL-12 (38–40). Upon activation, NK cells exert antiviral functions through both cytotoxic and cytokine-mediated mechanisms. NK cells eliminate infected cells via perforin- and granzyme-mediated cytotoxicity and secrete IFN-γ and TNF-α to suppress viral replication and recruit immune cells (37, 41). In addition to these effector functions, NK cells contribute to immune coordination through interactions with dendritic cells and adaptive immune populations (42, 43). Functionally, NK cell activity is further refined by the presence of distinct subsets, including immunoregulatory CD56^bright^CD16^−^ and cytotoxic CD56^dim^CD16^+^ populations, whose responses are governed by a balance of activating and inhibitory receptors (44). During chronic HCV infection, this balance becomes disrupted. Persistent antigen exposure and inflammation drive NK cell dysfunction, characterized by impaired cytokine production, altered receptor expression, and reduced antiviral coordination (45). Viral proteins such as NS5A downregulate activating receptors (e.g., NKG2D), while increased engagement of inhibitory pathways (e.g., NKG2A via HLA-E) further suppresses NK cell activity (46, 47). The hepatic microenvironment reinforces this dysfunction through immunosuppressive cytokines such as IL-10 and TGF-β (48). Additionally, viral factors including HCV-E2 interaction with CD81 directly inhibit NK cytotoxicity (49). Beyond these established mechanisms, recent evidence demonstrates that chronic HCV infection also induces functional skewing of NK cells toward increased cytotoxicity but reduced cytokine production, reflecting an imbalance between effector arms of innate immunity (50). This phenotype is particularly evident in FcϵRIγ^neg^ adaptive NK cell subsets, which exhibit increased PD-1 expression and impaired antibody-dependent cellular cytotoxicity (ADCC), especially in patients with advanced fibrosis (50). Importantly, NK cell dysfunction is not uniform and is influenced by disease stage. Patients with advanced fibrosis display heightened NK cell activation and exhaustion marker expression, whereas those with mild disease exhibit a phenotype closer to healthy controls, indicating heterogeneity in innate immune dysregulation (50). Together, these mechanisms impair effective viral clearance and promote persistence.

NK cell dysfunction is, however, only partially reversible. Following viral clearance with DAA therapy, NK cell phenotype and function undergo partial normalization, characterized by reduced PD-1 expression and improved IFN-γ production and ADCC capacity, particularly within adaptive NK cell population (50). Despite this recovery, normalization remains incomplete, and subset-specific defects persist, suggesting that prolonged antigen exposure imprints durable changes in NK cell function. These findings indicate that while DAA therapy alleviates key inhibitory pathways, it does not fully restore innate immune competence (50). In summary, innate immune responses initiate early antiviral defense against HCV, but viral evasion strategies and the tolerogenic liver environment undermine these mechanisms, enabling chronic infection and contributing to progressive liver disease. The mechanisms underlying interferon signaling are schematically depicted in **Figure 1**.

### Divergent innate immune sensing and interferon activation in HBV and HCV infection

HCV and HBV exhibit fundamentally distinct interactions with hepatocyte-intrinsic sensing pathways at the level of pathogen recognition and interferon induction. HCV replication generates cytosolic RNA intermediates that are accessible to RIG-I–like receptors, leading to MAVS-dependent activation of IRF3 and NF-κB and subsequent transcription of type I interferons. However, this signaling axis is rapidly attenuated by viral proteases such as NS3/4A, which cleave MAVS and disrupt mitochondrial antiviral signaling, thereby limiting IFN production despite ongoing viral replication (51). In contrast, HBV fails to induce a robust interferon response in hepatocytes despite active replication. This reflects a failure of effective pattern recognition rather than downstream blockade. HBV genomic replication occurs within nucleocapsid structures that physically shield viral nucleic acids from cytosolic sensors, preventing activation of RNA- or DNA-sensing pathways (52). In addition, hepatocytes exhibit limited DNA-sensing capacity due to deficient cGAS-STING signaling, further restricting innate immune activation during HBV infection (53).

HBV further reinforces this low-immunogenic state through active molecular mechanisms that suppress detection thresholds and interferon induction. Viral RNA intermediates undergo adenosine deaminases acting on RNA 1-mediated adenosine (A)-to- inosine (I) editing, which alters RNA structure and reduces recognition by RIG-I and MDA5, thereby preventing activation of IRF3 and downstream interferon transcription (53). In parallel, viral proteins such as HBx interfere with key signaling nodes including MAVS, TBK1, and STAT pathways, further suppressing interferon production and ISG induction (54). In contrast, HCV does not prevent initial sensing but instead permits partial activation of interferon pathways while disrupting signal propagation and effector responses. These differences define two mechanistically distinct immune evasion strategies: HBV minimizes interferon induction at the level of pathogen recognition, whereas HCV permits detection but suppresses signaling downstream of sensor activation. This divergence results in fundamentally different interferon landscapes, with implications for antigen presentation, inflammatory tone, and subsequent T cell priming and differentiation.

## Role of CD4^+^ and CD8^+^ T cells in hepatitis C virus infection

**Figure 2:**
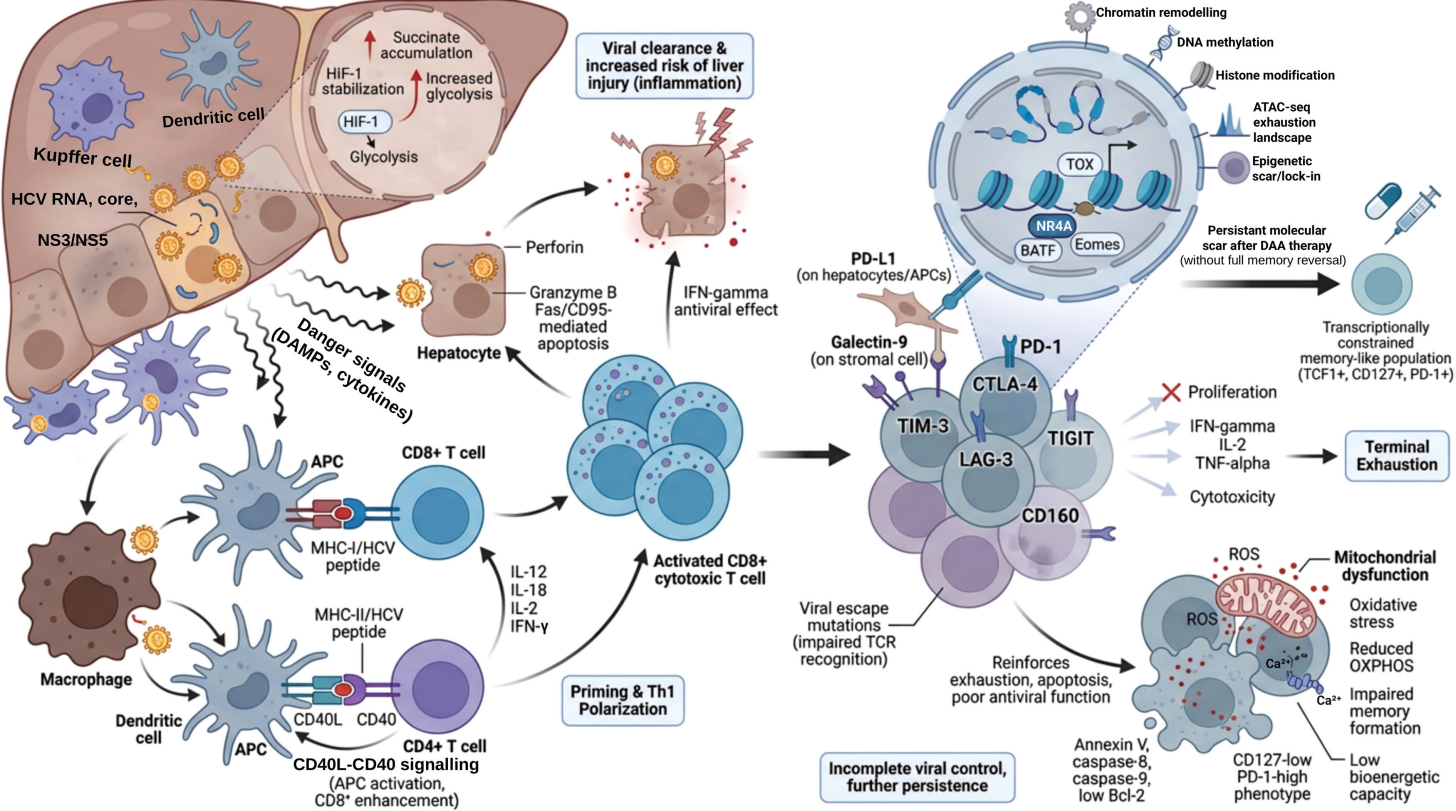
Integrated model of T cell activation, exhaustion, epigenetic imprinting, and metabolic dysfunction in chronic HCV infection. HCV infection of hepatocytes leads to the accumulation of viral RNA and proteins (core, NS3/NS5), triggering the release of danger-associated molecular patterns (DAMPs) and cytokines that are captured by liver-resident Kupffer cells, dendritic cells, and infiltrating APCs. These cells internalize viral material and present processed peptides via major histocompatibility complex (MHC) class I and II molecules, thereby activating CD8^+^ cytotoxic T lymphocytes and CD4^+^ T helper cells. CD4^+^ T cells provide essential help through IL-2 and IFN-γ secretion and CD40L-CD40-mediated licensing of APCs, promoting effective priming and Th1 polarization of CD8^+^ T cells. Activated CD8^+^ T cells eliminate infected hepatocytes through perforin- and granzyme-mediated cytotoxicity and Fas/CD95-dependent apoptosis, while also exerting non-cytolytic antiviral effects. However, persistent antigen exposure and viral escape mutations drive progressive T cell exhaustion characterized by upregulation of inhibitory receptors including PD-1, CTLA-4, TIM-3, LAG-3, TIGIT, and CD160, resulting in impaired proliferation, cytokine production, and cytotoxicity. Chronic stimulation induces stable epigenetic reprogramming mediated by TOX, NR4A, BATF, and Eomes, establishing a persistent molecular “epigenetic scar” that limits full functional recovery even after direct-acting antiviral therapy. Concurrent mitochondrial dysfunction, oxidative stress, and calcium dysregulation impair bioenergetic capacity, promote apoptosis, and reinforce T cell dysfunction, ultimately contributing to incomplete immune restoration despite viral clearance.

CD4^+^ T helper cells and CD8^+^ cytotoxic T lymphocytes (CTLs) represent the principal mediators of cellular antiviral defense. Specifically, CD8^+^ T cells orchestrate the elimination of infected hepatocytes, a process essential for viral clearance but one that simultaneously risks inducing substantial liver pathology if not strictly regulated (55). These cells eliminate pathogens through cytolytic pathways using perforin and granzyme, while simultaneously employing noncytolytic mechanisms to inhibit viral multiplication. Furthermore, the establishment of sustained, multiepitope-specific T cell responses is a prerequisite for spontaneous viral resolution, often relying on Fas/CD95-mediated apoptosis to excise infected hepatocytes from the liver parenchyma (**Figure 2**) (56). Conversely, the exhaustion of these T cell populations is a hallmark of chronic infection, often characterized by the upregulation of inhibitory receptors such as PD-1 and a progressive loss of effector functionality (57). This decline in functionality is exacerbated by the emergence of viral escape mutations that subvert TCR recognition, alongside a chronic inflammatory milieu that promotes T cell terminal differentiation and functional failure (58). Critical gaps remain in determining whether these exhausted T cell populations can be reinvigorated following direct-acting antiviral therapy or if permanent functional impairment persists in the absence of viral stimulus. Addressing this concern, emerging evidence suggests that although antiviral therapy effectively clears HCV and reduces immune-mediated liver injury, it does not fully reverse the metabolic and transcriptional reprogramming that underlies T cell dysfunction (59, 60). Consequently, persistent exposure to viral antigens drives progressive T cell differentiation and exhaustion, which may limit functional recovery even after viral clearance, highlighting the potential need for combinatorial immunomodulatory strategies targeting inhibitory receptors such as PD-1 and CTLA-4 to restore T cell competency (61).

The magnitude, breadth, and persistence of virus-specific T cell responses strongly influence whether HCV infection is spontaneously cleared during the acute phase or progresses to chronic infection accompanied by progressive liver disease. Although chronic infection often stems from viral evasion via epitope mutations that impair MHC class I presentation, immune-mediated damage frequently manifests as cirrhosis or hepatocellular carcinoma when persistent inflammation remains unchecked (9, 18). Beyond these mechanisms, the virus actively sustains its persistence by inducing Fas-mediated apoptosis of virus-specific immune cells while promoting recruitment of regulatory populations to the liver (62). Furthermore, sustained expression of inhibitory markers such as Tim-3, 2B4, and CD160 on liver-resident T cells facilitates a state of profound exhaustion, particularly when CD8^+^ T cells encounter conserved viral epitopes that limit escape mutations (63). These molecular changes are accompanied by downregulation of T-box transcription factors, reinforcing diminished cytotoxic potential and impaired polyfunctionality (64). The initiation of T cell-mediated immune responses occurs during the early phase of HCV infection following viral entry and replication within hepatocytes (**Figure 2**). Viral antigens derived from infected hepatocytes are captured, processed, and presented by antigen-presenting cells (APCs), including dendritic cells, macrophages, and Kupffer cells residing in the liver (65). These professional APCs activate naive T lymphocytes within lymphoid tissues through antigen presentation via major histocompatibility complex (MHC) molecules (66). Viral peptides presented through MHC class I molecules activate CD8^+^ cytotoxic T lymphocytes, whereas peptides presented through MHC class II molecules stimulate CD4^+^ T helper cells (67). Recognition of antigenic peptides by T cell receptors initiates signaling cascades leading to T cell activation, proliferation, and differentiation into effector cells (**Figure 2**).

CD4^+^ T helper cells play a fundamental role in coordinating adaptive immune responses against HCV (68). They mediate this through secretion of cytokines such as IL-2 and IFN-γ, which support the functional maturation and cytotoxic activity of CD8^+^ T cells (69). Moreover, CD4^+^ T cells provide essential co-stimulatory signals that prevent premature exhaustion of antiviral effectors (13, 70). In particular, early CD4^+^ T cell help mediated by CD40L signaling is critical for priming functional, liver-homing CD8^+^ T cell subsets and establishing effective antiviral immunity (71). Among CD4^+^ T cell subsets, Th1 cells are especially important in antiviral immunity. These cells promote viral eradication by facilitating the priming of CD8^+^ T cells through antigen-presenting cells, ensuring efficient antigen recognition and response (72). Failure of this CD4^+^ T cell-mediated crosstalk is a common precursor to viral persistence, leading to impaired dendritic cell function and suboptimal CD8^+^ T cell responses (13, 72). Furthermore, IL-12 production by dendritic cells promotes IFN-γ secretion from activated T cells, reinforcing antiviral polarization (73). This process is further amplified by Kupffer cells and macrophages, which release IL-12 and IL-18 to support differentiation of virus-specific CD4^+^ T cells into Th1 effectors (74). Additionally, CD4^+^ T cell-mediated licensing of dendritic cells via CD40L/CD40 interactions remains essential for optimal CD8^+^ T cell activation and function (**Figure 2**) (75). These coordinated interactions promote robust immune responses associated with viral clearance and improved treatment outcomes. However, the effectiveness of these responses is often limited by functional heterogeneity and transient sequestration of CD4^+^ T cells within infected liver tissue.

Clinical and immunological studies have demonstrated significant differences in T cell responses between individuals who clear HCV infection and those who develop chronic disease (76). Individuals who spontaneously resolve infection exhibit strong, broad, and multi-specific T cell responses targeting multiple viral antigens (76). These responses are sustained over time and are characterized by robust proliferation of both CD4^+^ and CD8^+^ T cells, along with production of antiviral cytokines such as IL-2 and IFN-γ (76, 77). Sustained CD4^+^ T helper cell activity is particularly critical for maintaining effective CD8^+^ CTL responses and long-term immune control (70, 71). In contrast, individuals who progress to chronic infection typically exhibit weak, transient, or narrowly focused CD4^+^ T cell responses that decline early during infection (77). Taken together, effective coordination between CD4^+^ and CD8^+^ T cell responses is essential for viral clearance in HCV infection. However, viral evasion mechanisms, immune exhaustion, and impaired T cell help disrupt this balance, promoting chronic infection and progressive liver disease.

### Compartmental differences in intrahepatic and peripheral T cell responses

Compartmentalization of HCV-specific T cell responses is driven by differential antigen exposure and tissue-specific signaling that impose distinct differentiation and survival programs in the liver compared to the periphery. In chronic infection, NS3-specific CD8^+^ T cells are markedly enriched within the liver, reaching 1.0–2.3% of intrahepatic CD8^+^ T cells, while remaining barely detectable in paired peripheral blood, indicating selective recruitment or retention at the site of infection (78). These intrahepatic cells uniformly express CD69, reflecting recent antigen encounter and sustained TCR engagement (78). This persistent antigen stimulation drives an activated effector phenotype but concurrently induces inhibitory pathways that limit function. Liver-infiltrating CD8^+^ T cells express high levels of PD-1 and low levels of CD127, a combination that reduces responsiveness to survival cytokines and promotes apoptosis through cytokine withdrawal-mediated activation of caspase-9 (79). As a result, these cells display impaired proliferation, diminished IFN-γ production, and defective cytotoxicity despite their activated state (79). In contrast, peripheral HCV-specific CD8^+^ T cells are exposed to lower antigen levels and maintain a resting memory phenotype with reduced activation marker expression and lower apoptotic susceptibility (79). This divergence is reinforced by trafficking dynamics, as CCR7^−^ effector-memory CD8^+^ T cells preferentially accumulate in the liver, whereas CCR7^+^ less differentiated populations remain in circulation (80). Consequently, the liver represents an antigen-rich but survival-constrained environment in which continuous TCR signaling, PD-1 engagement, CD127 downregulation, and apoptosis pathways converge to produce a numerically enriched yet functionally impaired CD8^+^ T cell compartment.

Intrahepatic CD4^+^ T cell responses are shaped by localized immunoregulatory networks that actively suppress effector function. CD4^+^FoxP3^+^ regulatory T cells accumulate within necroinflammatory regions of the liver and localize in close proximity to CD8^+^ T cells, enabling direct suppression through contact-dependent mechanisms (81). This suppression is reinforced by IL-10, which is produced by both CD4^+^ and CD8^+^ HCV-specific T cells and inhibits T cell proliferation and cytokine production. HCV-specific IL-10 responses arise early during acute infection and are associated with reduced CD4^+^ T cell proliferation, increased viral load, and elevated liver injury markers (81, 82). During chronic infection, IL-10 responses remain prominent and are broader than in resolved infection, whereas IFN-γ responses are diminished, indicating a shift toward regulatory dominance (82). In addition, the liver supports the expansion of IL-10-producing regulatory CD8^+^ T cells that suppress neighboring effector T cells, further limiting antiviral activity (80). These mechanisms operate alongside persistent antigen stimulation to maintain intrahepatic T cells in a state of partial activation with constrained effector output. In contrast, peripheral T cell responses are less influenced by these localized suppressive networks and are primarily governed by reduced antigen exposure, resulting in a less activated and less inhibited phenotype.

## T cell exhaustion and inhibitory receptors in chronic hepatitis C virus infection

Persistent antigen stimulation during chronic HCV infection leads to the development of T cell exhaustion, a state of progressive dysfunction characterized by reduced effector function, impaired proliferative capacity, and diminished antiviral activity. This phenotype is marked by sustained expression of inhibitory receptors such as PD-1, CTLA-4, and Lag-3, which function as immune checkpoints that restrain T cell activation and limit immunopathology (83). Constitutive expression of these receptors on HCV-specific CD4^+^ T cells is associated with early defects in proliferation and declining cell frequencies, often preceding progression to chronic infection. In contrast, CD8^+^ T cells, although similarly exhausted, tend to persist at higher frequencies, suggesting that CD4^+^ T cell loss may involve distinct mechanisms such as deletion or impaired differentiation into memory populations (84, 85).

In addition to PD-1, other co-inhibitory receptors, including TIGIT, further constrain T cell function, contributing to a complex inhibitory network that reinforces exhaustion (86). Persistent antigen exposure and the upregulation of inhibitory pathways such as CTLA-4 and TIM-3 act synergistically to suppress T cell proliferation and cytokine production (87). Evidence suggests that this functional decline is driven primarily by limited proliferative capacity rather than an intrinsic inability to produce cytokines, with eventual loss of T cells potentially mediated by apoptotic pathways involving PD-1 and Fas signaling (88). Moreover, the co-expression of multiple inhibitory receptors, such as PD-1 and Tim-3, on intrahepatic HCV-specific T cells further amplifies functional impairment by reducing antigen-driven proliferation and IFN-γ production (15, 63). This exhaustion phenotype is particularly pronounced within the intrahepatic microenvironment, where elevated levels of inhibitory ligands such as PD-L1 and Galectin-9 reinforce T cell dysfunction and promote apoptosis of virus-specific cytotoxic T lymphocytes (89). Such regional differences reflect the distinct metabolic and cytokine environment of the liver, which further shapes T cell fate. The progression toward terminal exhaustion has been described as “antigen addiction,” whereby continuous antigen exposure sustains the survival of dysfunctional T cell populations (90). Following the removal of chronic antigen stimulation through direct-acting antiviral (DAA) therapy, HCV-specific T cells may undergo partial functional recovery; however, they often retain a persistent molecular “scar” that distinguishes them from fully functional memory T cells (91). These epigenetic and transcriptional imprints limit the restoration of polyfunctional cytokine production, including IL-2 and TNF-α, even when inhibitory receptor expression declines (92, 93). Although proliferative capacity may improve after viral clearance, persistent epigenetic remodeling suggests that complete functional restoration is rarely achieved (94).

Recent transcriptomic and epigenetic studies further support this concept, demonstrating that exhaustion is associated with a stable, antigen-dependent transcriptional program that persists even after viral eradication (95, 96). Chromatin accessibility analyses reveal that the exhaustion-associated epigenetic landscape undergoes only limited remodeling following DAA therapy, indicating a durable state of differentiation (97). This “epigenetic lock-in” is reinforced by transcription factors such as TOX, which act as molecular regulators sustaining the exhausted phenotype even in the absence of antigen stimulation (98, 99). Consequently, effective therapeutic strategies may need to target both inhibitory receptor signaling and underlying epigenetic programs to restore T cell function. In parallel with functional exhaustion, increased susceptibility to apoptosis further contributes to the decline of virus-specific CD8^+^ T cells during chronic HCV infection. Early during infection, only a subset of these cells produces cytotoxic molecules such as granzyme B and perforin, and this capacity diminishes over time (100). Many virus-specific CD8^+^ T cells undergo programmed cell death, leading to a progressive reduction in their numbers. This susceptibility is associated with high PD-1 expression and reduced levels of anti-apoptotic molecules such as Bcl-2, which are essential for T cell survival (101, 102).

Intrahepatic CD8^+^ T cells appear particularly vulnerable to apoptosis compared with circulating T cells. Both activation-induced cell death (AICD), mediated by death receptor signaling and caspase-8 activation, and antigen-independent cell death (ACAD), involving intrinsic mitochondrial pathways and caspase-9 activation, contribute to this process (103). Notably, while PD-1 expression is commonly associated with T cell exhaustion and apoptosis, emerging evidence indicates that PD-1 signaling can also confer resistance to restimulation-induced cell death (RICD), thereby supporting the survival of activated T cells under certain conditions (104). Nevertheless, in chronic infection, elevated PD-1 expression generally correlates with increased apoptotic susceptibility, as indicated by enhanced Annexin V expression and reduced survival signaling (79). Specifically, CD127**^–^**PD-1^hi^ T cell subsets exhibit reduced Bcl-2 expression and increased caspase-8 activity, suggesting that terminal differentiation is closely linked to apoptotic deletion (105). Co-expression of additional inhibitory receptors such as TIM-3 and LAG-3 further amplifies apoptotic signaling, contributing to progressive loss of functional T cells (106). During periods of high viral replication, CD8^+^ T cells expand but often display a dysfunctional phenotype characterized by high PD-1 and low CD127 expression, rendering them prone to apoptosis (107). This is further compounded by the loss of memory-associated markers, preventing differentiation into long-lived memory T cells capable of sustaining protective immunity (108, 109). Consequently, the persistence of highly apoptotic, PD-1^+^ CD8^+^ T cells limit the formation of a durable and self-renewing memory T cell compartment (79). In summary, chronic antigen exposure in HCV infection drives a multifaceted program of T cell exhaustion, characterized by inhibitory receptor signaling, epigenetic imprinting, and increased apoptotic susceptibility, collectively limiting effective antiviral immunity and long-term immune protection.

### Metabolic-epigenetic coupling in exhausted HCV-specific T cells

Chronic antigen stimulation induces coordinated metabolic and epigenetic reprogramming that stabilizes the exhausted state in HCV-specific CD8^+^ T cells (91). Early in infection, exhaustion-committed T cells undergo a metabolic shift characterized by reduced glucose uptake, diminished glycolytic capacity, and impaired mitochondrial respiration (110). This is accompanied by downregulation of glucose transporters such as GLUT1 and reduced extracellular acidification rate, indicating impaired glycolytic flux. Mitochondrial dysfunction is further showed by decreased oxidative phosphorylation, loss of membrane potential, and elevated production of reactive oxygen species (110). These alterations are actively regulated through signaling pathways involving ATM and p53, which suppress glycolysis and promote oxidative stress, thereby constraining bioenergetic capacity required for proliferation and effector differentiation (110). In parallel, inhibitory receptor signaling attenuates PI3K/AKT/mTOR activity, further limiting nutrient uptake and anabolic metabolism (91). Together, these processes establish a bioenergetic constraint that restricts T cell expansion and effector function. Notably, mitochondrial dysfunction persists after viral clearance, indicating that metabolic reprogramming is maintained independently of antigen load and contributes to sustained functional impairment (111).

Metabolic alterations are mechanistically coupled to epigenetic remodeling that enforces a stable transcriptional program of exhaustion. Genome-wide chromatin accessibility analyses demonstrate that exhausted HCV-specific CD8^+^ T cells acquire a distinct epigenetic landscape characterized by persistent accessibility at regulatory regions controlling exhaustion-associated genes, including *TOX*, *HIF1A*, and inhibitory receptor loci (97). This chromatin architecture undergoes limited remodeling following DAA-mediated viral clearance, resulting in retention of “epigenetic scars” that sustain repression of effector programs despite reduced antigen stimulation (110). Metabolic state reinforces this configuration by regulating substrate availability for chromatin-modifying enzymes, including histone methyltransferases (HMTs), which drive repressive chromatin remodeling and global gene downregulation in late-stage exhaustion (110). Functional studies demonstrate that pharmacological inhibition of these epigenetic regulators restores mitochondrial function and antiviral activity, indicating that metabolic and epigenetic pathways operate as an integrated regulatory axis (110). Together, these findings support a model in which metabolic insufficiency and epigenetic fixation act in concert to stabilize T cell dysfunction, thereby limiting immune restoration even after elimination of persistent viral replication.

## Epigenetic modifications of HCV-specific T cells

HCV infection is associated with profound epigenetic reprogramming of virus-specific T cells, which contributes to the establishment and maintenance of T cell exhaustion. Persistent antigen exposure during chronic infection induces stable changes in chromatin accessibility, DNA methylation, and histone modifications that collectively reshape transcriptional programs governing T cell function. These epigenetic alterations are not merely secondary consequences of exhaustion but actively reinforce the dysfunctional state by restricting the expression of genes associated with effector function, proliferation, and memory differentiation (98, 112). In HCV-specific CD8^+^ T cells, exhaustion is characterized by a distinct epigenetic landscape that differs fundamentally from that of functional effector or memory T cells (97). Genome-wide chromatin profiling studies have demonstrated that exhausted T cells acquire a unique set of accessible regulatory regions enriched for transcription factors such as TOX, NR4A, and BATF, which drive and stabilize the exhausted phenotype (99, 113). These factors act as key epigenetic regulators that integrate chronic TCR signaling into long-term transcriptional adaptations. This molecular signature persists as a “chronic scar” within the HCV-specific T cell compartment even after the complete cessation of viral replication. Notably, while memory-like TCF1^+^CD127^+^PD1^+^ populations persist post-therapy, they retain transcriptomic signatures of exhaustion, specifically elevated Eomes and PD-1 expression, that prevent them from fully transitioning into the robust, functional memory subsets observed after spontaneous viral clearance (105, 114). This suggests that the therapeutic elimination of HCV antigen is insufficient to fully erase the epigenetic footprints imprinted during chronic infection, thereby precluding the restoration of a truly naive-like functional state (91). Furthermore, researchers should prioritize investigating the intrahepatic immune environment and the epigenetic imprinting of immune cells, as this could determine whether selective targeting of chromatin-modifying mechanisms enhances the plasticity of these memory-like populations. For instance, the persistence of viral antigen stimulation leads to progressive remodeling of enhancer landscapes, resulting in the loss of accessibility at loci associated with cytokine production, including *Il2* and *Tnf*, while maintaining accessibility at exhaustion-associated genes such as *Pdcd1* (97, 98, 113). These epigenetic changes establish a transcriptional hierarchy in which inhibitory pathways dominate over effector responses, thereby locking T cells into a state of reduced functionality. Importantly, this reprogramming is reinforced by alterations in metabolic pathways, as exhausted T cells exhibit impaired mitochondrial function and reduced bioenergetic capacity, further constraining their ability to mount effective antiviral responses (59). Collectively, these findings indicate that epigenetic modifications serve as a fundamental mechanism by which chronic HCV infection enforces durable T cell dysfunction.

Notably, the epigenetic imprinting of exhausted T cells persists even after successful viral clearance, representing a major barrier to full immune restoration following direct-acting antiviral (DAA) therapy (115). Although antigen removal can partially restore T cell proliferation and reduce inhibitory receptor expression, the underlying chromatin landscape remains largely fixed, preventing complete reversion to a functional memory state (91). Studies using chromatin accessibility assays such as ATAC-seq have shown that only a limited subset of exhaustion-associated regions undergo remodeling after antigen clearance, while the majority of epigenetic features remain stable (97, 98). This phenomenon, often referred to as “epigenetic scarring,” reflects the long-term imprint of chronic antigen exposure and restricts the plasticity of T cells even in the absence of ongoing viral (116–118). Furthermore, single-cell transcriptomic analyses of liver-resident T cells in HCV-infected individuals have revealed the persistence of exhaustion-associated transcriptional signatures, including sustained expression of TOX and inhibitory receptors, despite viral eradication (95). These data suggest that exhaustion is not simply a reversible functional state but rather a differentiation program stabilized by epigenetic mechanisms. The maintenance of this program is further supported by DNA methylation patterns at key regulatory loci, such as *Pdcd1*, which remain demethylated and transcriptionally active even after antigen withdrawal (119, 120). Consequently, therapeutic strategies aimed solely at removing antigenic stimulation may be insufficient to fully restore T cell function. Instead, combinatorial approaches targeting both inhibitory receptor signaling and epigenetic regulators may be required to reverse exhaustion and re-establish effective antiviral immunity. Emerging evidence suggests that epigenetic-modifying agents, in combination with immune checkpoint blockade, may enhance the reinvigoration of exhausted T cells by reopening chromatin at effector loci and restoring transcriptional flexibility (98, 121). However, the potential risks of disrupting stable epigenetic programs, including the induction of immunopathology, necessitate careful evaluation. In summary, epigenetic modifications represent a central and enduring mechanism underlying T cell dysfunction in chronic HCV infection, with significant implications for both immune recovery and the design of next-generation immunotherapeutic strategies.

## Mitochondrial dysfunction and oxidative stress in HCV-Induced T cell impairment

Mitochondria are central regulators of cellular bioenergetics, redox homeostasis, and immune cell function. In antiviral immunity, particularly within T lymphocytes, mitochondrial integrity is essential for sustaining metabolic flexibility, cytokine production, proliferation, and long-term memory formation (59). During chronic hepatitis C virus infection, viral proteins profoundly disrupt mitochondrial function, leading to oxidative stress and metabolic dysregulation that impair T cell responses and promote viral persistence (122, 123). Several HCV proteins, including core and non-structural proteins, partially localize to mitochondria and mitochondrial-associated membranes. These interactions trigger enhanced calcium influx into mitochondria, elevating intramitochondrial Ca^2+^ concentrations. This stimulates nitric oxide production and inhibits respiratory chain complex I (122) progressively compromising oxidative phosphorylation, mitochondrial membrane potential, and electron transport chain activity (123). As electron transport becomes inefficient, electrons leak from the respiratory chain and react with molecular oxygen to produce excessive reactive oxygen species, a hallmark of HCV-induced mitochondrial dysfunction (124). Under physiological conditions, low ROS levels serve as signaling molecules regulating immune responses and metabolism. However, chronic HCV infection overwhelms cellular antioxidant systems, creating a persistent oxidative environment (125). This oxidative stress damages mitochondrial DNA, lipids, and proteins, further exacerbating dysfunction. In T cells, it disrupts activation and effector signaling pathways, reducing antiviral immunity (125). Interestingly, while OXPHOS impairment limits T cell bioenergetics, HCV-infected hepatocytes often maintain or even increase ATP levels through compensatory metabolic adaptations (126). Cells expressing HCV proteins exhibit enhanced glycolytic flux, enabling ATP production via non-oxidative glucose metabolism in a “Warburg-like” shift (126). This reprogramming supports infected cell survival despite mitochondrial damage (**Figure 2**). The key driver of this shift is HIF-1α, which upregulates glycolytic enzymes and glucose transporters (**Figure 2**). In HCV infection, HIF-1α stabilizes and accumulates in the nucleus of both experimental models and infected liver tissues, independent of hypoxia (126). Mechanistically, HCV-induced mitochondrial dysfunction disrupts the tricarboxylic acid cycle, accumulating metabolites like succinate, oxaloacetate, and pyruvate. These inhibit prolyl hydroxylase domain enzymes, preventing HIF-1α hydroxylation and proteasomal degradation under normoxic conditions (126). Stabilized HIF-1α translocates to the nucleus, heterodimerizes with HIF-1β, and drives transcription of glycolytic and survival genes.

Oxidative stress amplifies this pathway by depleting antioxidants like glutathione, which impairs ascorbate regeneration and PHD activity (124). This creates a feed-forward loop reinforcing glycolysis and mitochondrial dysfunction, fostering an environment conducive to viral replication while impairing immune function (123). In T cells, mitochondrial metabolism underpins differentiation and effector responses: activated effectors use both respiration and glycolysis, while memory cells rely predominantly on oxidative metabolism (59). HCV-induced impairment disrupts this balance, limiting antiviral activity (59, 125). Elevated ROS activates stress pathways promoting exhaustion, marked by reduced cytokines, proliferation, and upregulated inhibitory receptors (59, 125). It also damages mitochondrial membranes, releasing pro-apoptotic factors that trigger cell death or dysfunction. Collectively, these changes weaken antiviral immunity and contribute to chronic infection. Persistent damage may also drive long-term pathology like fibrosis and hepatocellular carcinoma via inflammation and genomic instability (123). By manipulating host metabolism to sustain ATP for replication despite mitochondrial injury (123), HCV compromises immune cell function (59). Thus, targeting mitochondrial function, redox balance, or HIF-1 signaling, potentially combined with epigenetic and checkpoint therapies, offers promising strategies to restore immunity and curb disease progression in chronic HCV.

## Immune restoration after HCV cure: mechanisms and therapeutic strategies

DAA therapy has reframed HCV from a chronic viral disease into a model for testing whether removal of persistent antigen is sufficient to restore antiviral immunity. The answer appears to be incomplete and biologically heterogeneous. DAAs efficiently eliminate viral replication by targeting NS3/4A protease, NS5A, or NS5B polymerase, but viral clearance does not uniformly reset the exhausted immune compartment (127). Recent study shows partial immune restoration, including reduced PD-1 expression on CD4^+^ T cells and improved proliferative capacity of HCV-specific CD4^+^ and CD8^+^ T cells after DAA-mediated cure (128). However, cytokine production by HCV-specific T cells, including IFN-γ and IL-2, may remain impaired despite viral eradication, indicating that proliferative recovery and effector recovery are mechanistically separable (128). This distinction is important because it challenges the assumption that antigen withdrawal alone reverses exhaustion. A systematic review of 26 studies involving 919 individuals concluded that most exhausted T cell subsets recover only partially after DAA cure, with advanced fibrosis associated with sustained exhaustion across multiple immune compartments (129). Thus, immune recovery after HCV cure is not a binary process but is shaped by fibrosis stage, duration of antigen exposure, pre-treatment differentiation state, and the depth of transcriptional or epigenetic imprinting.

Mechanistically, the strongest explanation for incomplete immune restoration is that chronic antigen exposure remodels HCV-specific CD8^+^ T cells beyond reversible checkpoint expression. After DAA cure, terminally exhausted CD127^low^ HCV-specific CD8^+^ T cells decline, while TCF-1^+^CD127^+^PD-1^+^ memory-like cells persist, producing an apparent memory polarization of the antiviral T cell pool (91). However, these retained memory-like cells do not fully resemble bona fide memory cells generated after spontaneous resolution. They continue to express exhaustion-associated programs involving TOX, EOMES, RUNX3, and BATF, indicating a persistent molecular scar of chronicity (91). Epigenetic studies extend this concept by showing that exhaustion-associated chromatin accessibility is only partially remodeled after cure, with scarred regulatory regions near *TOX* and *HIF1A* persisting long after antigen removal (97). This provides a mechanistic basis for weak long-term immune memory after DAA cure and may explain why cured individuals remain susceptible to reinfection. Importantly, antigen recognition intensity also appears to shape this scar, since HCV-specific CD8^+^ T cells targeting conserved epitopes show a stronger exhaustion program than those targeting escaped variant epitopes (91). We therefore propose that the quality of post-cure immune memory is determined not only by whether antigen is removed, but by how long and how strongly each T cell clone experienced antigen before cure.

These findings support a shift from nonspecific immune boosting toward mechanism-guided immune repair. Checkpoint blockade remains attractive because PD-1/PD-L1 and Tim-3 blockade can increase HCV-specific CD8^+^ T cell proliferation, IFN-γ production, IL-2 secretion, and in some settings cytotoxic function *in vitro* (130). Yet checkpoint blockade alone is unlikely to fully restore antiviral immunity when exhaustion is maintained by metabolic failure and epigenetic restriction. In early exhaustion, HCV-specific CD8^+^ T cells show impaired glycolytic and mitochondrial programs linked to ATM and p53 signaling, while late exhaustion is associated with broad transcriptional repression and metabolic dysfunction (110). Pharmacological targeting of p53, ATM, AMPK, p38, and histone methyltransferases can improve glucose uptake, reduce PD-1 expression, and restore IFN-γ, TNF-α, and IL-2 production in dysfunctional HCV-specific CD8^+^ T cells (110). These data argue that future immunotherapy should not rely on checkpoint blockade alone but should combine checkpoint release with metabolic and epigenetic reprogramming to reopen exhausted chromatin states and restore effector competence.

Therapeutic vaccination after DAA cure should also be reconsidered in light of these mechanisms. Vaccines that merely expand pre-existing HCV-specific memory-like CD8^+^ T cells may amplify scarred populations with limited cytokine and recall capacity. A more rational strategy would pair conserved-antigen vaccination with interventions that increase epigenetic plasticity, improve mitochondrial fitness, and selectively expand TCF-1^+^ progenitor-like cells before terminal exhaustion is fixed. This approach may be especially relevant for patients with advanced fibrosis, in whom immune restoration is least complete after DAA cure (129). Clinically, DAAs reduce fibrosis-related disease burden and lower the risk of HCC, decompensation, and mortality, but they do not eliminate all downstream consequences of chronic immune remodeling (131). The central knowledge gap is therefore no longer whether HCV can be cured virologically, but whether durable antiviral immunity can be rebuilt after years of antigen-driven exhaustion. Addressing this gap will require longitudinal studies that integrate TCR clonality, chromatin accessibility, metabolic profiling, fibrosis stage, and reinfection outcomes to identify which immune defects are reversible, which are fixed, and which are therapeutically druggable.
